# A Microcirculatory Theory of Aging

**DOI:** 10.14336/AD.2019.0315

**Published:** 2019-06-01

**Authors:** Kunlin Jin

**Affiliations:** Department of Pharmacology & Neuroscience, University of North Texas Health Science Center, TX 76107, USA

**Keywords:** aging, theory, microcirculation, impairment, lifespan

## Abstract

Aging is the progressive decline of physiological functions necessary for survival and reproduction. In gaining a better understanding of the inevitable aging process, the hope is to preserve, promote, or delay healthy aging through the treatment of common age-associated diseases. Although there are theories that try to explain the aging process, none of them seem to fully satisfy. Microcirculation describes blood flow through the capillaries in the circulatory system. The main functions of the microcirculation are the delivery of oxgen and nutrients and the removal of CO_2_, metabolic debris, and toxins. The microcirculatory impairment or dysfunction over time will result in the accumulation of toxic products and CO_2_ and loss of nutrition supplementation and O_2_ in corresponding tissue systems or internal organs, which eventually affect normal tissue and organ functions, leading to aging. Therefore, I propose a microcirculatory theory of aging: aging is the process of continuous impairment of microcirculation in the body.

One of the main goals in medicine today is the preservation of the quality of life, especially in the elderly. Ideologies such as “optimal”, “successful”, and “healthy” aging consist of the maintenance of cognitive and physical functions, continuous active engagement in daily activities of living, and the prevention of deleterious age-related diseases and disabilities [1]. Thus, we hope not only to live longer, but also healthier. To achieve these, we ought to understand the process of aging.

Pursuing a set of criteria for successful aging goes beyond considering intellectual points of view; the elucidation of underlying mechanisms for the decline in bodily functions can spur us to find innovative solutions for age-associated diseases such as stroke, Alzheimer’s, and cancer. In the larger scope of things, this will mean decreased healthcare costs and caregiver burden in the long run as a larger proportion of elderly people will be living longer and healthier lives. Yet, the current proposed theories of aging that fit into two broad categories: programmed or error theories, do not seem to fully satisfy or explain the aging process [1]. Programmed theories postulate that aging follows an intrinsic timetable that may be a continuation of the process that controls growth and development from childhood. On the other hand, error theories propose that the aggregated damage from the environment to different levels of the biological system directly causes aging. Importantly, regardless of our genetic heritage, our bodies are constantly calibrating themselves through complex biological reactions, some of which can cause damage, and thus aging. Exchange of materials, supplements, O_2_ and fluids etc. are essential for these complex biochemical reactions, which, interestingly, depend on the microcirculatory function.

Microcirculation describes blood flow through the capillaries in the circulatory system. The main functions of the microcirculation are the delivery of oxygen and nutrients and the removal of CO_2_, metabolic debris, and toxins. The microcirculatory impairment or dysfunction over time will result in the accumulation of toxic products and CO_2_ and loss of nutrition supplementation and O_2_ in corresponding tissue systems or internal organs, which eventually affect normal tissue and organ functions, leading to aging.

Our healthspan depends on the functional quality of these various systems (tissue and organ). However, our lifespan depends on the service life of each system, and the individual service life depends on the microcirculatory function in that system. Therefore, aging is driven by the continuous impairment of the microcirculation.

## 1. Microcirculation overview

### 1.1 Definition of the Microcirculation

The term “microcirculation” in contrast with *macrocirculation* (which is the flow of blood to and from organs), refers to a network of terminal vessels comprising arterioles, capillaries and venules that are <100 μm in diameter. In other words, the microcirculation is defined as the blood flow through the smallest vessels in the vasculature and are embedded within organs and tissues, which facilitate the exchange of biological material between the blood and tissue via its large surface area and low blood velocity in these regions. For organs to function well, there must be sufficient perfusion throughout the tissue in the form of intact and appropriate microcirculatory vascularization. Apart from these blood vessels, lymphatic capillaries and collecting ducts are also included, but are not included in this discussion [2].

### 1.2 Structure of the *Microcirculation*

Although the microcirculatory architecture can vary between tissues, the basic elements that make up the microvasculature networks are similar. According to Spalteholz and Krogh, the microcirculation can start off with large arterioles that then connect with smaller arterioles followed by capillaries and venules that are organized similarly to the arterioles, resembling [3]. This arrangement resembling a tree-like network comprise of microvessels categorized by their anatomical features and direction of blood flow from pre-capillaries (arterioles and precapillary sphincters) to capillaries then post-capillary venules [3-5].

Arterioles’ diameters are often <500 μm and are made up of two to four smooth muscle cells arranged circumferentially to form an external muscular coat. Precapillary or terminal arterioles are one layered smooth muscle cells that form thin branches of arteries measuring only ~10-20 μm in diameter. They belong to the arterial side of the microcirculatory system and their primary roles include delivering blood to localized tissues and regulating the rate of blood delivery. Additionally, walls of large arterioles are made up of a single endothelial cell layer (known as tunica intima) and are surrounded by an internal elastic tissue followed by a multilayered tunica media consisting smooth muscle cells [6]. Terminal arterioles in contrast, only have a single layer of smooth muscle cells.

Ultrastructurally, most microcirculatory vessels are lined with flattened endothelial cells making up the endothelium and surrounded by pericytes that are contractile by nature. The endothelium not only plays an important role for regulating water and dissolved substances across the interstitial space but also allows blood to flow uninterruptedly because of its smooth surface. Precapillary sphincters (comprised of 1-2 layers of contractile smooth muscle) can also be found surrounding the entrances of the capillaries to adjust the flow of blood into capillaries [7].

Downstream from the terminal arterioles is the tree-like network of capillaries, which are much denser than the arteriolar network because of its occasional cross connections and parallel branches space about 20-30 μm apart [8]. These capillaries are the smallest blood vessels in the body with diameters ranging from 5 to 10 µm. They structurally resemble a tube made of endothelial cells, have very thin walls and are surrounded by a basement membrane. Pericytes can be found on almost all capillaries including terminal arterioles and postcapillary venules [9]. They regulate the flow of blood and thus, blood pressure by contracting and decreasing the size of the arterioles. Similar to the arterioles, the capillary endothelium regulates the exchange of water and dissolved substances and provides uninterrupted blood flow. In the event of a leak or injury to the capillary, its endothelium can produce clotting factors to allow the blood to form a clot and seal the leak.

Post-capillary venules are ~10 µm in diameter and structurally similar to capillaries. Smooth muscle cells are absent in small venous vessels (<30 µm in diameter) in skeletal muscle but are become increasingly evident as the diameter of the venous vessels increase [9]. The network of venules runs parallel to the arterioles and drains blood from the capillaries. It can store up to 75% of total blood volume, making it a large reservoir of low pressure for returning blood to the heart. Venules play important roles in immunity, the exchange of macromolecular substances, and regulation of post-capillary vascular resistance.

Considering the microcirculatory network in its totality, the number of microvessels in the body and itself as an organ is extremely large, including about 2×10^9^ capillaries. Based on the vessels’ individual structural characteristics, and thus functionality, the highly interconnected and continuous network consisting branches that run parallel to each other still manages to reflect considerable differences. Arteries carry blood that feed into arterioles and capillaries then to venules, veins, and ultimately back to the heart. Thus, there seems to be a significant degree of organization within this system.

### 1.3 Function of the *Microcirculation*

The primary function of the circulatory system is to exchange substances between blood and tissue, and these exchange processes take place in the microcirculation, which provides tissue perfusion, fluid homeostasis, and delivery of oxygen and other nutrients. It also controls temperature and the inflammatory response.

#### 1.3.1 Oxygen exchange

The microcirculatory system is special in that it is the primary mode of exchange for substances, such as oxygen and cell waste amongst others, through the endothelial cells’ phospholipid bilayers.

For a long time, oxygen loss has been assumed to occur only in the capillaries according to Krogh’s cylinder model [10], which is used extensively for microcirculatory oxygen exchange studies. In tissues such as the mesentery and resting skeletal muscle, low metabolic rates instead cause oxygen loss to occur in the arterioles [11]. Therefore, oxygen exchange in microcirculatory vessels can take place in a convective (in arterioles) or diffusive (in capillaries) manner [12]. So far, reports have documented the ability of oxygen to diffuse not only between a single capillary and an organ when there is sufficient PO_2_ gradient but also between various pairs of microvessels [13].

#### 1.3.2 Fluid exchange

The microcirculation is essential for the exchange of fluids between blood and tissue. According to Starling’s hypothesis, fluid exchange based on transcapillary filtration depends on the balance of pressure gradients of the four Starling forces: hydrostatic pressure in the capillary, hydrostatic pressure in the interstitium, oncotic pressure in the capillary, oncotic pressure in the interstitium. Starling’s equation for fluid exchange in capillaries and postcapillary venules also considers surface area and hydraulic conductivity of the endothelial membrane [14].

#### 1.3.3 Solute exchange

The microcirculatory network is also crucial for immunological defense, delivering hydrophilic solutes and molecules such as vitamins and hormones to organs, and bulk delivery of substances between organs for synthesis or storage. Exchange of solutes occur via bulk flow of fluid, transcytosis, and diffusion [15]. Particularly, passive diffusion provides a quick and highly efficient form of exchange over short distances of ~10 μm between cells and the blood supply.

#### 1.3.4 Inflammation and leukocytes transmigration

Apart from the movement of gas, fluids, and solutes across the endothelium, white blood cells are also able to move from the blood to tissue in a process termed transmigration, which is an important feature of microvascular function[16]. Transmigration or extravasation begins with the white blood cell approaching then rolling along the endothelium, it then adheres to the selectins and integrins expressed on endothelial cells of the post-capillary venules and larger venules (as large as 50 μm), and is pulled through the endothelium into the interstitium and finally migrates to the site of injury or infection through a chemotactic gradient [17].

## 2. Microcirculatory impairment with aging: where is the evidence?

### 2.1 Age-related capillary loss

There is a substantial number of studies presenting strong evidence of decreased vessel density with age, indicating an age-associated failure of vascular recovery in organs such as the brain in animals and humans alike.

In studies involving aged rodents: healthy senescent rats (29 months) experienced a loss of about 40% of arteriolar density on the cortical surface compared with young adult rats (13 months) [18]. There was also 39% fewer arterioles penetrating the cortex and cortical surface [18-21]. Arteriole-arteriole anastomoses and venules were also subject to similar decreases, indicating that aging can affect vessels found on the surface and even the deeper cortical layers of the brain. In the hippocampus of aged rats, there was a 20% decrease in capillary number, 3% decrease in capillary length, and 24% increase in intercapillary distance [22]. Comparable reductions could also be found in other brain regions including the brain stem, cortex, and white matter [22-28]. Villena et al. [29]demonstrated that rats (18-24 months) had decreases in the number of capillaries (9%), volume of capillaries (19%), and length of capillaries (11%) however, for older rats aged 24-28 months, these proportions increased by 7%, 15%, and 9%, respectively. Although there were no significant differences in capillary[30] and vessel [31] densities, one study still found decreases in capillary number and length with respect to increasing age [30], which conflicts with another report by Bar that saw a 16% increase in vessel length [32]. With respect to age-associated capillary loss in muscle such as cardiac and skeletal muscle, average diameter did not change significantly but segmental length experienced and increase in the microvasculature of the cremaster muscle in adult (12 months) and senescent F344 rats [33]. Similarly, in mice, reported no significant difference in vessel density was found [34]. More often than not, decreases in capillary and arteriolar densities are the most prominent features in the terminal vascular bed of the aging murine myocardium [35].

In studies involving aged humans: Capillary density decreased by 16% in the calcarine cortex [36-38] while vascular density decreased by 50% in the paraventricular nucleus [39], frontal cortex [40], and putamen [41] with no change in the supraoptic nuclei [39] and the cortex [40, 41]. White matter vascular density also decreased in subjects between 57-90 years [42] but another report found no change [43]. Interestingly, a study [44, 45] found that subjects ranging from 64 to 74 years experienced increased vessel density *vs.* young subjects, while those >75 years had similar vessel density *vs.* the young. However, capillary length and volume for the 65-74 and 75-94 age groups were found to be decreased. Disorganization of the branching geometry in arterioles and capillaries of the skin[46], skeletal muscle[47, 48], and retina[49] can also be an effect of aging. Importantly, angiogenesis has been found to be impaired in aged tissues[50, 51], which could contribute to the significant decreases in vascular density and number that has been reported.

### 2.2 Age-related decreases in blood flow

Having adequate blood flow to tissues is critical for ensuring their proper function and health. A breakdown in or obstruction to this flow can lead to decreased metabolic capacity and consequently, impaired tissue function and ultimately failure. Continuous and sufficient blood flow (CBF) is vital to cell function; thus, tissue perfusion, typically quantified by measuring the volume of blood passing through the microvascular network in a given volume of tissue over a certain duration, is a key indicator of organ or tissue health. Since regional blood flow strongly affects cellular metabolic capacity, it is then crucial for us to unravel the inherent intricacies in microvessel characteristics and reactivity that change with age for understanding the progressive deterioration of tissue function due to age-related impaired metabolic activity.

Although much *in* vivo data is available with regards to microcirculatory structure and function, most were performed in lower animals [52]. In 1912, Warren Lombard first described human capillary circulation in the nail fold where he discovered the presence of capillaries by applying glycerin on the skin and observing it under the microscope with strong reflected light [53]. Since then, human microcirculatory studies have been limited to viewing microcirculation on or very close to the surface of organs e.g., skin and retina, using epi-illumination. A more recent innovation known as orthogonal polarization spectral imaging (OPS) is used as it significantly improves contrast *vs*. epi-illumination. For a better sense of blood flow in microvessels, it is now possible to combine microscopy to determine P_O2_ using a surface electrode. Alternatively, laser Doppler flowmetry can be utilized as a stand-alone to assess microvessel function as it is able to measure both the flux and velocity of red blood cells [54]. Blood flow to the skin has been shown to significantly decrease as we age, indicating an inverse relationship. A study showed that between the ages of 20 and 70, the estimated loss could be up to 40%. Moreover, blood flow to the skin was significantly reduced with increases in total cholesterol levels and systolic blood pressure. Correlation analysis showed strong associations between skin blood flow and age with systolic blood pressure being the least associated [55]. Recently, it was reported that there was a ~25% decrease in basal blood flow to the leg in old *vs.* young healthy sedentary men, suggesting a diminished limb vascular conductance. Further analysis showed that the decreased blood flow was associated with reduced limb oxygen demand [56]. Laser speckle contrast imaging (LSCI) data revealed that there is a substantially higher complexity in young *vs.* old individuals [57]. Other studies have confirmed the occurrence of reduced vascular conductance and blood flow to the leg while exercising [58] and experiencing reactive hyperemia [59] in old *vs.* young subjects, and could be attributed to increased vasoconstriction with age [60]. Interestingly, there are conflicting reports concerning how age affects blood flow. In comparing elderly *vs*. young persons, there does not seem to be any differences in basal blood flow [61], post exercise hyperemia [61], and blood flow responses after exercising a single limb [62, 63]. Such discrepancies may arise from the recruitment of active or sedentary individuals [59, 60]. Moreover, it is unclear whether blood flow changes due to increasing age were a consequence of decreased cardiac output while exercising or diminished local vascular capacity [64].

Conventional perfusion imaging methods such as positron-emission tomography (PET), single-photon emission computed tomography (SPECT), X-ray computed tomography (CT) and contrast-enhanced MRI has been used to measure cerebral blood flow [33]. In recent years, arterial-spin labelling (ASL) is increasingly becoming the mainstream perfusion imaging method of choice as it boasts minimal invasiveness requiring no exogenous tracers. More novel methods based on ASL such as continuous ASL (CASL) and pseudo-CASL have also been introduced in the clinic. Using a slew of imaging methods, when comparing old *vs*. young subjects, cerebral blood flow (CBF) was reported to be substantially decreased [65-67] and decreasing CBF was found to correlate with increasing age [68]. Of note, CBF changes with age may occur as early as middle age and appears to have regional differences [69]. In a study examining how age affects CBF in cognitively healthy old persons, advanced anatomical models and pulsed ASL along with high-resolution structural MRI discovered significant CBF reduction with age and this was seen particularly in the cortex. These results corroborate with those found in PET and MRI studies [70, 71] as well as reports that used ASL previously [72, 73]. Importantly, CBF decline agreed with cross-sectional and longitudinal CT findings especially in the frontal, temporal, and parietal brain regions [74]. Meltzer et al [75] and others [59,76] found decreases in mean cortical CBF with respect to healthy aging using PET, but these were not significant after correcting for partial-volume effects arising from individual variations in cerebral volume differences. PET simulations on images acquired from brain MRI of healthy subjects from 18 to 79 years of age predicted a similar result; there was a negative association between age and CBF in grey matter even after controlling for brain tracer concentration, but the correlation ceased to exist after adjusting for partial-volume effects [77].

In studies involving rodents and non-human primates, age-associated decline in regional CBF (rCBF) have also been reported. For instance, comparing 12-month old and 24-month old F344 rats, rCBF was significantly reduced mainly in the posterior brain with a mean decline of 17%, which could be related to partial functional deafferentation [78]. Changes to regional cerebral metabolic rate of glucose consumption (rCMRGlc) and rCBF in aged monkeys were associated with age, but rCBF and rCMRGlc coupling was still preserved even with age [79]. Indeed, the underlying mechanisms regulating blood flow into and through the capillary bed are many, including vascular architecture, arteriolar reactivity and vessel density [80], all of which could be jeopardized as we age.

## 3. Aging: consequence of microcirculatory impairment

The saying by Thomas Sydenham, “A man is as old as his arteries” [81], holds true even more so in this day and age as factors of vascular aging are reported to be closely associated with chronological age. Indeed, alterations in vascular mechanics and structure are related with vascular aging, resulting in less elastic arteries and diminished arterial compliance. Furthermore, the increased diffusion distance for oxygen caused by reduced capillary numbers and density, gives rise to heterogeneous perfusion [82], where the close proximity of perfused capillaries and non-perfused capillaries triggers alterations to oxygen extraction even when blood flow to the tissue is conserved [83]. Under normal physiological conditions, the microcirculatory blood flow is adapted to the metabolic levels of human tissues and organs, so the physiological functions of various organs in the human body can function as they should. Once the microcirculation of the human body is impaired, cells would not be able to get enough nutrition and oxygen, and meanwhile, CO_2_ and metabolic products, including those that are toxic, cannot be removed and will accumulate. Consequently, deterioration of physiological functions of cells and then organs that are necessary for survival and reproduction will occur. *Microcirculatory* impairment arises in adulthood and becomes progressively impaired with aging; the corresponding tissue system or internal organs are affected and unable to function normally, which eventually lead to aging. Therefore, aging is the process of continuous impairment of microcirculation in the body.
